# Supplementary material for: Impact of the COVID-19 Pandemic on Staging Oncologic PET/CT Imaging and Patient Outcome in a Public Healthcare Context: Overview and Follow Up of the First Two Years of the Pandemic
Source: Cancers (Basel). 2023 Nov 10;15(22):5358. doi: 10.3390/cancers15225358 (PMC10670509; doi:10.3390/cancers15225358)
Supplement: Supplementary file 1 [file cancers-15-05358-s001.zip › cancers-2646122-supplementary.pdf]

Supplemental Figures

Figure S1 – Kaplan Meyer Curve for Gender

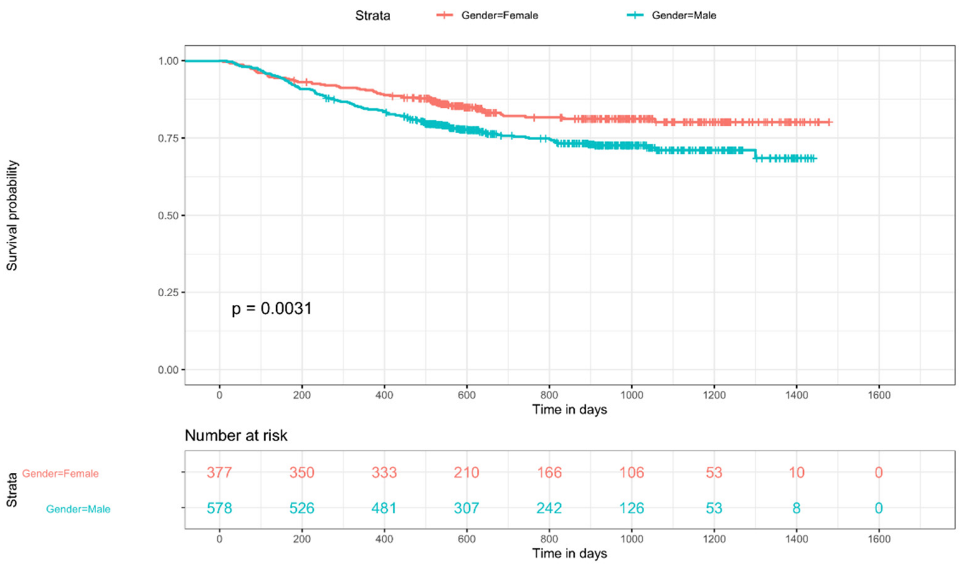

Figure S2 - Kaplan Meyer Curve for Type of Cancer

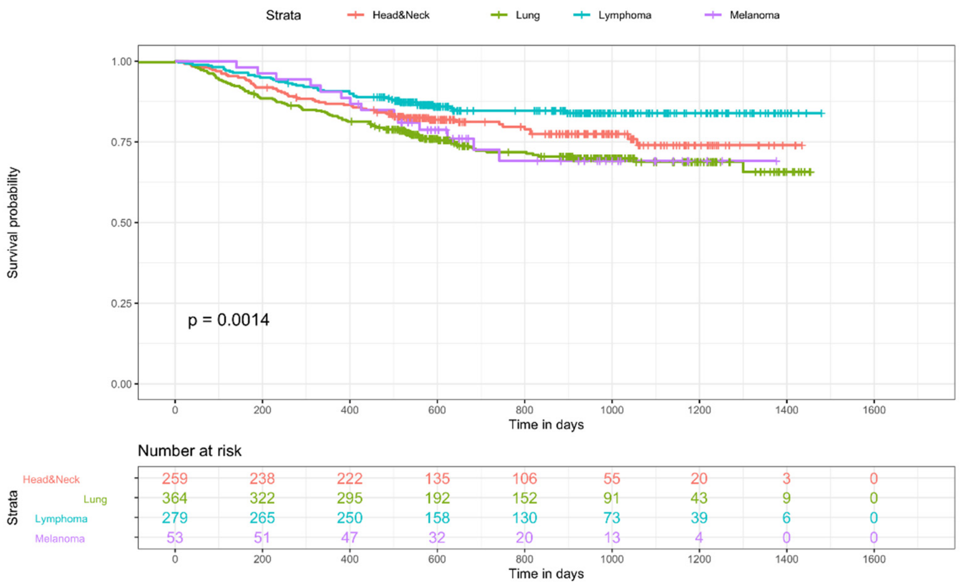

Figure S3 - Kaplan Meyer Curve for Cancer Stage

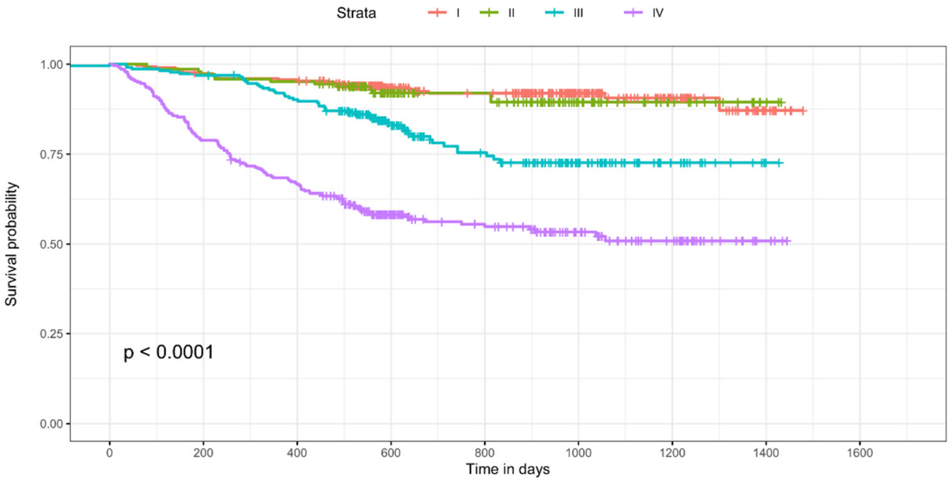

Strata	0	200	400	600	800	1000	1200	1400	1600
I	301	292	286	196	170	95	44	9	0
II	145	141	138	87	73	37	16	3	0
III	224	218	201	121	82	47	17	2	0
IV	279	220	184	111	81	51	28	4	0
